# IT’S BETTER TO GIVE THAN RECEIVE, OR IS IT? THE EFFECT OF SOCIAL SUPPORT ON THE PSYCHOLOGICAL WELL-BEING OF OLDER PARENTS

**DOI:** 10.1093/geroni/igz038.1819

**Published:** 2019-11-08

**Authors:** Erik Blanco

**Affiliations:** California State University Los Angeles, Los Angeles, California, United States

## Abstract

The longevity revolution has led to more years of shared lives between older parents and adult children. Having these extra years together can be offset by the stressful life transitions of widowhood, health declines, and increased level of disability experienced by older parents. These transitions can lower older parents’ psychological well-being. Although social support to/from adult children has the potential to buffer these effects, most older parents wish to remain independent, even in later life, making them reluctant to accept social support from their adult children. The purpose of this paper is to examine whether the provision or receipt of social support between older parents and adult children, influences positive mood and negative mood. Secondary data on older adults (n = 461) with adult children who participated in the 2004 wave of the LSOG were used. The results revealed that the provision of social support by older parents to adult children significantly increased parents’ positive mood showing that it is better to give than receive. The results for the receipt of social support were more complex. Results suggest that when someone has a higher level of disability and does not receive social support their negative mood increases, but when someone has a high level of disability and does receive social support there is no effect on negative mood. This proposes that the receipt of social support is particularly important when the parent is in need of support and it is better to receive than give when parents are in need.

